# Severe coarctation of the aorta diagnosed during pregnancy: the role of multimodal imaging and multidisciplinary approach to a complex subject—a case report

**DOI:** 10.1093/ehjcr/ytag041

**Published:** 2026-01-24

**Authors:** Débora Sá, Rita Salgueiro Neto, Filipa Reis, João Adriano Sousa

**Affiliations:** Cardiology Department, Hospital Central do Funchal, Avenida Luís de Camões no 57, Funchal, Madeira 9004-514, Portugal; Obstetrics and Gynecology Department, Hospital Central do Funchal, Avenida Luís de Camões no 57, Funchal, Madeira 9004-514, Portugal; Obstetrics and Gynecology Department, Hospital Central do Funchal, Avenida Luís de Camões no 57, Funchal, Madeira 9004-514, Portugal; Cardiology Department, Hospital Central do Funchal, Avenida Luís de Camões no 57, Funchal, Madeira 9004-514, Portugal

**Keywords:** Adult congenital heart disease, Coarctation of the aorta, Maternal cardiology, Case report

## Abstract

**Background:**

Coarctation of the aorta (CoA) is a well-known congenital heart disease, which is often associated with other cardiac and vascular anomalies, most frequently a bicuspid aortic valve. Aortic coarctation is an unusual cause of hypertension during pregnancy, and its management is not clarified.

**Case summary:**

We report a case of a 24-year-old pregnant woman with long-standing hypertension, who presented at the end of the first trimester with severe refractory hypertension. The diagnostic investigation culminated in the diagnosis of a CoA. Facing risks for pursuing pregnancy such as aortic complications, hypertensive disorders, and foetal adverse outcomes, aside from limited therapeutic options and having in mind the relatively early stage in pregnancy, it was decided by multidisciplinary team and the patient for pregnancy interruption. After that, the patient was treated by percutaneous dilatation of aortic coarctation with stent implantation, with good results. Recently, the patient was able to carry out a newly full-term pregnancy without major incidents.

**Conclusion:**

Coarctation of the aorta should be considered in pregnant women with refractory hypertension. It is associated with considerable risks to the mother and to the foetus. In cases presenting with refractory severe hypertension, termination of pregnancy needs to be considered, especially in first-trimester pregnancies.

This case also highlights the importance of investigating secondary causes of hypertension in young people, as these can often be treated, averting potentially severe complications.

Learning points

- Severe native coarctation diagnosed during pregnancy carries very high maternal–foetal risk (mWHO IV) and requires urgent multidisciplinary management.
- In early pregnancy with severe or refractory hypertension, counselling should include continuation of pregnancy with deferred stenting or the option of medical termination.
- Persistent hypertension in young adults should always prompt evaluation for secondary causes, enabling timely diagnosis of CoA and prevention of major complications.

## Introduction

Coarctation of the aorta (CoA) is the sixth most common congenital heart defect, accounting for 5–8% of cases.^1^ It frequently coexists with other cardiac anomalies, such as a bicuspid aortic valve, and with extracardiac vascular anomalies, including the anomalous origin of the right subclavian artery and intracerebral aneurysms.^2^ While typically diagnosed and managed in childhood, up to 20% of cases present in adulthood, frequently accompanied by chronic hypertension.

Very rarely, CoA may be diagnosed during pregnancy, posing substantial risks to the maternal-foetal dyad.^3^ Pregnancy is categorized as an intermediate risk in the setting of repaired CoA (mWHO 2.0 II–III) and high risk in those with severe (re)coarctation (mWHO 2.0 IV), but prospective data to validate this risk classification are scarce.^1, 2^

Surgical resection is recommended for neonates and young children, while transcatheter treatment is preferred for adults and older children.^4, 5^ However, due to paucity of data, guidelines lack specific recommendations for managing CoA with refractory hypertension during pregnancy.

## Summary figure

**Figure:**
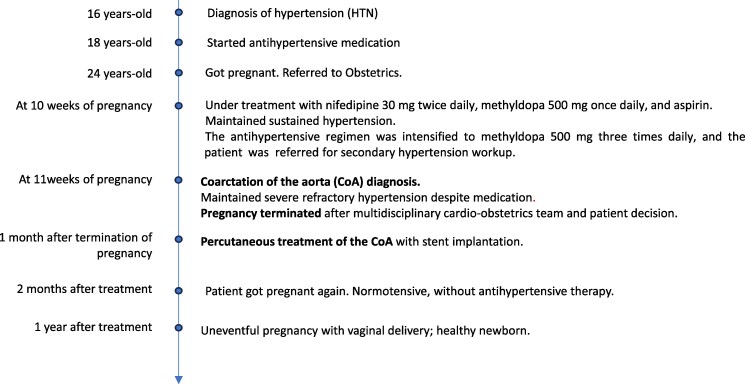


## Case presentation

We present a case of a 24-year-old woman with a history of hypertension since adolescence and a thyroidectomy for Graves' disease presented during pregnancy with difficult-to-control hypertension. She does not recall having a specific work-up for secondary causes of arterial hypertension. No relevant family history was reported.

At 11 weeks of pregnancy, despite therapy adjustments, she maintained severe hypertension. Physical examination revealed a systolic blood pressure (BP) of 158 mmHg in the right arm and 143 mmHg in the right leg with normal cardiopulmonary auscultation.

**Figure 1:**
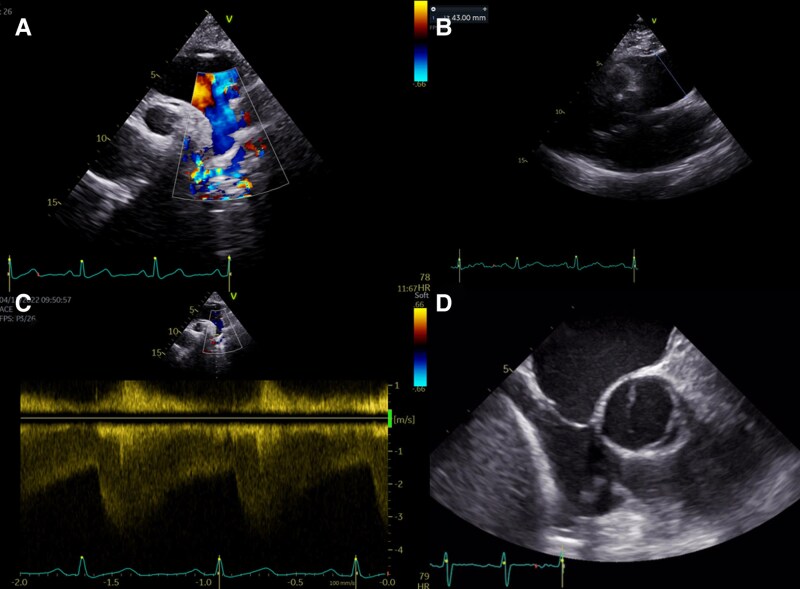
Echocardiographic findings. *(A*) Suprasternal window echocardiogram revealing turbulent flow in the descending thoracic aorta. *(B*) Transthoracic echocardiogram demonstrates a dilated ascending aorta measuring 43 mm. *(C*) Continuous Wave Doppler at the coarctation site exhibiting the characteristic ‘sawtooth’ pattern. *(D*) Transoesophageal echocardiogram reveals a Siervers type 1 bicuspid aorta.

These findings prompted an investigation into secondary hypertension. The 24-hour urine test was negative, and the ECG was normal. The transthoracic echocardiogram showed a coarctation of the descending thoracic aorta with a maximum gradient of 63 mmHg. Additionally, a bicuspid aortic valve was observed without functional impact, along with a dilated ascending aorta with a maximum diameter of 43 mm and a mild concentric hypertrophy of the left ventricle with preserved ejection fraction. Transoesophageal echocardiogram confirmed a *Sievers* type 1 bicuspid aortic valve with fusion of the right and left coronary cusps. (*Figure 1*)

**Figure 2:**
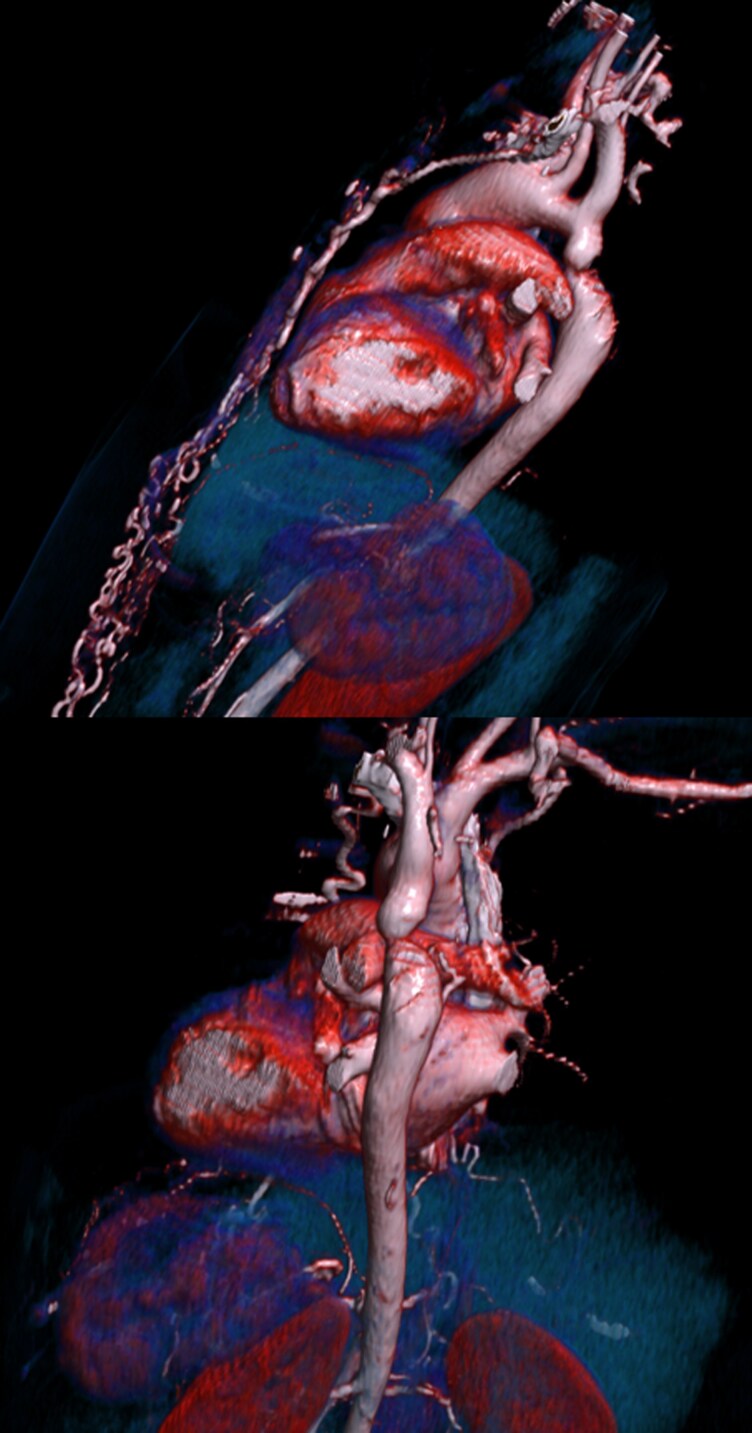
3D reconstruction from thoracic angioCT angiography showing the region of aortic coarctation and the exuberant collateral circulation through intern mammary.

The thoracic CT angiography confirmed the CoA, closed to ligamentum arteriosus, 15 mm distal to the left subclavian artery, with 3 mm of caudal extension and 16, 9, and 23 mm of luminal diameter before, at the coarctation site, and after it, respectively. (see Supplementary material online, *Video S1*) It causes ingurgitation of the innominate artery, left common carotid artery, and left subclavian artery, and an exuberant collateral circulation through internal mammary, vertebral, and posterior intercostal arteries, the latter being responsible for inferior rib border notching (*Figure 2*). Descending thoracic post-coarctation and abdominal aorta showed no alterations. CT angiography of the brain excluded arteriovenous malformations.

The complex nature of these findings motivated a multidisciplinary approach where a cardio-obstetrics team—including specialists in Obstetrics, Cardiology, and Interventional Pediatric Cardiology—deliberated on the risks of pursuing pregnancy and the best management options. Two possible strategies were presented to the patient: (1) continuation of pregnancy, trying to maintain strict blood pressure control, hospital bed rest, and stent implantation after 1–3 weeks (awaiting completion of the first trimester); or (2) pregnancy termination followed by percutaneous stent implantation under safer conditions. After thorough counselling and discussion of the prognosis, the patient and her family made an informed decision to pursue medical termination of pregnancy (TOP) due to the potential risks to the mother's health. The patient was closely monitored throughout the process of TOP, and expulsion of the foetus was achieved safely. The patient was discharged after receiving psychological care.

One month after, a cardiac catheterization was performed, showing a peak-to-peak gradient of 39 mmHg and confirming a haemodynamically significant CoA. The patient was treated by percutaneous dilatation of the coarctation with covered stent implantation 8/3.4 cm, BIB 16/4.5X9/9 inflated at 5Atm (see Supplementary material online, *Video S2*), with good angiographic and hemodynamic results (residual gradient transcoarctation of 0 mmHg). The patient was discharged two days after, sustaining normal BP without antihypertensive therapy.

Eighteen months post-procedure, the patient remains normotensive under no medication. She recently achieved a full-term pregnancy and had an uncomplicated vaginal delivery of a healthy newborn. Both mother and child were doing well at the last follow-up.

## Discussion

Pregnancy hormonally-mediated vessel wall weakness and increased thrombogenicity can exacerbate CoA-associated vasculopathy, significantly increasing maternal risk of cerebrovascular events and aortic rupture or dissection, hypertension crisis and congestive cardiac failure.^3^ While risk classifications exist, they primarily rely on limited retrospective cohorts, and clinical guidelines are predominantly founded on expert opinions and limited data.^4^

Severe native CoA during pregnancy is classified as mWHO 2.0 risk class IV, corresponding to an extremely high risk of maternal mortality or severe morbidity. With a CARPREG II score of 2, the estimated maternal cardiac event rate is around 70%.^1,2^ According to the *2025 ESC Guidelines for the Management of Cardiovascular Disease and Pregnancy*, termination of pregnancy should be considered and discussed in women with mWHO 2.0 class IV risk due to the exceptionally high maternal and foetal risk.^1^

Historically, women with CoA were believed to have higher risk for hypertensive disorders of pregnancy such as preeclampsia compared to general population (20% vs. 8%). However, recent studies of women with repaired CoA had few serious complications with a strong correlation to pre-pregnancy antihypertensive use, suggesting the risk may be lower than previously thought.^5^

Ramlakhan *et al*.^6^ describe a maternal major adverse cardiac event (MACE) rate of 4.3% and a hypertensive disorders of pregnancy rate of 5.3% in a population of 303 pregnant women with CoA, which are not more prevalent than in the general population; however, only 9,6% involved unrepaired CoA, and the limited prevalence of preexisting hypertension or antihypertensive medication suggests that this cohort mainly involves mild unrepaired CoA or pseudocoarctation, which was not the case for our patient.

Another study showed that a minimum aortic diameter correlates with adverse cardiovascular events in pregnancy. A 12 mm threshold has the best discriminatory capacity for predicting such events.^7^ In our case, the minimum diameter was 9 mm, indicating a substantial risk of cardiovascular complications.

Furthermore, important foetal complications secondary to systemic hypertension may arise, such as foetal growth restriction and preterm delivery (as a result of placental abruption or ischaemia).^8^

In cases where medical management is insufficient, percutaneous stent placement should be considered, ideally after the first trimester and in specialized centres. This is a very rare procedure in pregnancy, with less than ten cases described in the literature, most of them performed after the second trimester of pregnancy,^9–11^ and only one in the first trimester in an emergency context.^8^

These procedures are performed under fluoroscopy, entailing ionizing radiation exposure. As the risk is highest during organogenesis and decreases thereafter, the ESC recommends deferring percutaneous interventions until after 12 weeks of gestation. Nevertheless, maternal safety must always take precedence and should ultimately guide the clinical decision.^1^

Facing these risks and the early stage of pregnancy, the informed decision of TOP was made by the patient. Definitive treatment was successfully performed thereafter, allowing the patient to pursue another pregnancy with a significantly lower risk.

Hypertension frequently complicates the long-term follow-up of CoA patients, despite successful repair and regardless of the type of correction performed. Several mechanisms may contribute, including upregulation of the renin–angiotensin system, impaired vasoreactivity, aortic arch geometry abnormalities, baroreflex dysfunction, and abnormal aortic distensibility. Despite its relevance, no specific guidelines exist. Lifelong follow-up is required, with annual clinical assessment, ECG, and echocardiography.^12^

Our case highlights the diagnostic value of meticulous physical examination and echocardiographic assessment in young hypertensive individuals. An appropriate investigation of secondary causes of hypertension in the past could have spared the patient from this burden and mitigated the cardiac risks associated with pregnancy.

## Patient perspective

**‘**When I received the diagnosis, I felt fear, confusion, and sadness all at once. The medical team carefully explained everything—the risks for the baby and me, the possible outcomes, and the treatment options. It was not an easy decision, especially knowing I would have to be transferred to another city, remain hospitalized, and undergo an invasive procedure that could affect my child. In the end, I chose the safer path for my own health. Even though it was one of the hardest decisions of my life, I felt respected, informed, and cared for. Looking back now, I believe that the empathy and honesty of the team were just as important as the medical treatment itself. And most importantly, after the treatment, I was able to experience a much safer pregnancy.’

## Lead author biography


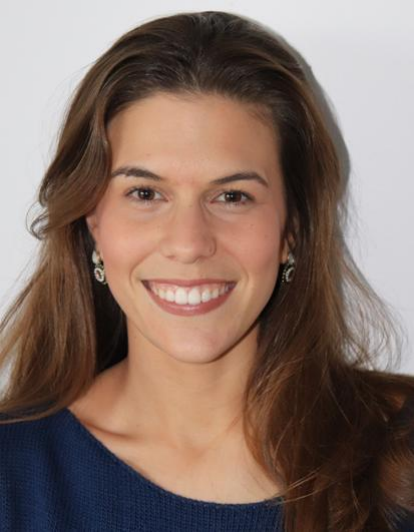


Rita Salgueiro Neto graduated in Medicine in 2016 from the Faculty of Medicine at the University of Lisbon and is currently completing her final year of residency in Obstetrics and Gynecology in Portugal. She has developed a strong interest in obstetric ultrasound and prenatal diagnosis, with a particular focus on optimizing maternal=E2=80=93fetal health through advanced imaging techniques. Beyond her clinical training, she is committed to research and academic work aimed at enhancing diagnostic precision and contributing to better outcomes in maternal and foetal medicine.

## Supplementary Material

ytag041_Supplementary_Data

## Data Availability

The data underlying this article are available in the article and its online Supplementary material.

## Notes

**Consent:** The author confirms that written consent for submission and publication of this case, including images, has been obtained, in accordance with COPE guidelines.
