# Supplementary material for: Severe coarctation of the aorta diagnosed during pregnancy: the role of multimodal imaging and multidisciplinary approach to a complex subject—a case report
Source: Eur Heart J Case Rep. 2026 Jan 24;10(2):ytag041. doi: 10.1093/ehjcr/ytag041 (PMC12924163; doi:10.1093/ehjcr/ytag041)
Supplement: ytag041_Supplementary_Data [file ytag041_supplementary_data.zip › Video Legends.docx]

**Video 1:** 3D reconstruction from thoracic angioCT angiography displaying the region of aortic coarctation.

**Video 2:** Percutaneous dilatation of aortic coarctation with covered stent implantation 8/3.4cm, BIB 16/4.5X9/9 inflated at 5Atm.
